# Role of a novel mouse mutant of the *Galnt2*^*tm*1*Lat*/*tm*1*Lat*^ gene in otitis media

**DOI:** 10.3389/fneur.2022.1054704

**Published:** 2023-02-16

**Authors:** Weijun Ma, Heng Li, Juan Hu, Ying Gao, Hui Lv, Xiaotong Zhang, Qing Zhang, Min Xu, Ying Cheng

**Affiliations:** ^1^Department of Otolaryngology-Head and Neck Surgery, Second Affiliated Hospital of Xi'an Jiaotong University, Xi'an, Shaanxi, China; ^2^Department of Otorhinolaryngology, Shiquan County Hospital, Ankang, Shaanxi, China; ^3^Department of Otolaryngology-Head and Neck Surgery, Xinhua Hospital Affiliated to Shanghai Jiao Tong University School of Medicine, Shanghai, China

**Keywords:** genetic susceptibility, otitis media, mutant *Galnt2* homozygote, hearing loss, mouse model

## Abstract

Genetic susceptibility is one of the most important causes of otitis media (OM). Mutant *Galnt2* homozygote (*Galnt2* ^tm1Lat/tm1Lat^) mimics human otitis media in comparable pathology and causes hearing loss. Otitis media is characterized by effusion and dysregulated mucosa proliferation and capillary expansion in the middle ear cavity, which is associated with hearing loss. The mucociliary dysfunction could be seen in the middle ear cavity (MEC) in a patient harboring the disease that develops in severity with age by a scanning electron microscope. Tumor necrosis factor alpha (TNF-α), transforming growth factor-beta 1 (TGF-β1), Muc5ac, and Muc5b upregulate the expression in the middle ear, which correlates with inflammation, craniofacial development, and mucin secretion. The mouse model with a mutation in the *Galnt2* (*Galnt2* ^tm1Lat/tm1Lat^) was explored in this study as a novel model of human otitis media.

## Introduction

Otitis media (OM) is the main cause of hearing loss among children, which is featured primarily as an inflammation of the middle ear mucosa (1). A marked association of sequelae and complications is observed following OM with hearing loss. The OM-related hearing loss is reported to affect 30.82 per 10,000 patients, with ~21,000 people dying annually from the complications of OM (2). Human susceptibility to OM is a multifactor process, including the participation of the adaptive and native immune systems, dysfunction of the Eustachian tube, the invasion of a viral load and bacteria, and the participation of genes and environment (3). A growing body of evidence is available to suggest that heredity is an important risk factor for OM (4, 5). The animal models of OM with defined gene defects offer significant value for the study of the disease course and pathogenesis of the OM (6). Tumor necrosis factor alpha (TNF-α) plays a key role in initiating and maintaining inflammatory responses in various diseases. In the OM, the homeostatic imbalance of TNF-α is the main cause of its chronic inflammation. The upregulation of TNF-α is always evident in the OM (7). Genetic defects leading to the upregulation of TNF-α provide insights into the construction of the OM models.

*GALNT2* is a gene on chromosome 1q42 within ~150 kb of the lead single-nucleotide polymorphism (SNP), which is located in an intron of the gene. It belongs to the N-acetyl galactosamine (GalNAc)-transferase family transferring an N-acetyl galactosamine to the hydroxyl group of a serine/threonine residue at the start of O-linked oligosaccharide biosynthesis (8). This glycosylation plays an important role in insulin resistance diabetes and chronic inflammation (9, 10). In addition, polypeptide N-acetylgalactosaminyltransferase 2 (GALNT2) knockout has been demonstrated to significantly upregulate the TNF-α levels through a disintegrin and metalloprotease (ADAM)-mediated ectodomain (10).

Exon 7 deletion in Galnt2 mouse was originally intended to be elaborated in a study on the lungs, but it was unexpectedly discovered that mice have otitis media. This study aimed to analyze the ear to determine whether otitis media was an isolated case or widespread in mutants. The present study hypothesized that if OM were prevalent in Galnt2 mutants, it would probably be due to the upregulation of TNF-α that caused chronic inflammation in the middle ear. It was also found that mice with this mutation develop otitis media from birth and exhibit progressive hearing loss, effusion of the middle ear cavity, and upregulated TNF-α and transforming growth factor beta (TGF-β) in the middle ear. In addition, malformation and dysfunction of the Eustachian tube, dysregulated mucosa proliferation and capillary expansion, and the mucociliary impairment are all found to lead to the incidence of OM in the *Galnt2* homozygous mutant mice.

## Materials and methods

### Mouse husbandry and genotyping

Galnt2^tm1Lat/tm1Lat^ homozygous mice were obtained from and bred at the Wolstein Animal Research Facility of Case Western Reserve University. Sixty-four homozygous Galnt2^tm1Lat/tm1Lat^ mutant and 60 wild type mice were used in the study. Mice were raised in a ventilated room with 12-h light/dark cycle and free access to food at 21°C. Mice <7 days were genotyped by PCR, the experimental protocol was approved by the Health Sciences Institutional of Animal Care center and Ethics Committee of Case Western Reserve University (approval numbers: 2008-0174 and 2008-0156) and Second Affiliated Hospital of Xi'an Jiaotong University (approval number: 2019-268). PCR primers used for tail snip genotyping are presented as follows:

P1: GGTCCTGACCTTCCTAGACAGTCACTGC

P2: GCACTCTCCAAGGGCATGACAGAGC

P3: GGGGGAGGATTGGGAAGACAATAGC

The mice used in this study (termed Galnt2^tm1Lat/tm1Lat^) were constructed, initially characterized, and provided by Dr. Lawrence A. Tabak, Section on Biological Chemistry, National Institute of Dental and Craniofacial Research, NIH, to Dr. Q. Zheng while he was on the faculty of CWRU. Details about the construction and characterization of these mice by Dr. Tabak and collaborators (reported as Galnt2^−/−^ mice) may be found in Verzijl et al. (11).

**Figure 1:**
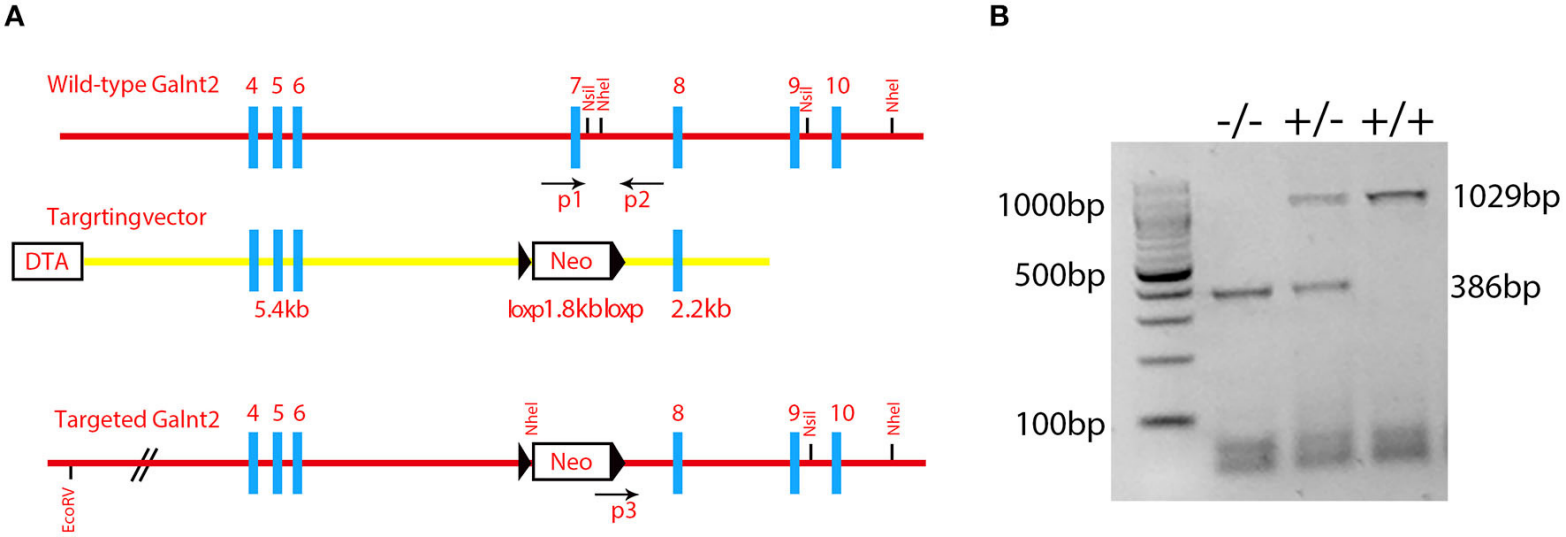
Identification of the mutant mice. **(A)** A schematic diagram of the *Galnt2* targeting vector, *Galnt2* genomic sequence, and the targeted allele. Gray rectangles represent exons; bold blue lines represent the region homologous to the *Galnt2* genomic sequence; black triangles represent *lox*P sites. Neo, neomycin positive selection cassette; DTA, diphtheria toxin negative selection cassette. P1, P2, and P3 indicate genotyping primers. A Neo cassette with flanking *lox*P sites and homology mini-arms cloned into the Galnt2 sequence to replace the exon 7 that contains the amino acid residues DSHCEC, crucial for Galnt2 catalytic activity. **(B)** Genotyping of Galnt2 variants. The wild-type mice (+/+) show one band (1029 bp), the mutant mice (-/-) show one band (386 bp), and the heterozygous mice (+/-) show two bands (386 bp and 1029 bp).

All information in Figure 1 was derived from data provided by Dr. Tabak and his colleagues at NIDCR, NIH. However, neither Dr. Tabak nor any member of his lab were involved in the conduct or interpretation of any remaining experiments reported in this paper. We had difficulty breeding these mice, and therefore did not use conventional back crossing prior to performing the analyses they reported.

### A comprehensive evaluation of hearing impairment

Wild-type and mutant mice were anesthetized with avertin (0.5 mg per gram of mice). The procedure was performed at normal temperature in a quiet room. A computer-aided evoking system (Intelligent Hearing Systems, Miami, FL, USA) was used to measure the auditory brainstem response (ABR) and distortion product otoacoustic emission (DPOAE) amplitudes of mice, as reported earlier (11). The present study also utilized a tympanometer from Maico Diagnostics (Maico Co, Ltd., Berlin, Germany) to measure the condition of the tympanic membrane (12). The measurement data were described by normal distribution and recorded by a two-way analysis of variance (ANOVA) with Bonferroni's *post-hoc* test.

### Observation of the middle ear and the structure of the adjacent skull base

Both wild-type and Galnt2^tm1Lat/tm1Lat^ mutant mice at the age of 2 months were killed by CO_2_ asphyxiation (*n* = 6 in each genotype, including three male and three female mice). The angle from the middle line of the skull base and the bony component of the Eustachian tube were measured for both genotypes. The tympanic membrane and the skull base were examined, and pictures were taken with a microscope (Leica S6D, Leica Microsystems, Wetzlar, Germany). The ImageJ software (Java 1.6.0-20 (32-bit) NIH, USA) was applied for angle measurement. The measurement data were analyzed by Student's *t-*test.

### Bacterial culture and identification

Four mice at the age of 3 months in each group were killed by CO_2_ asphyxiation. The middle ear of the mice was isolated and washed with sterile saline. After shaking, the water was inoculated onto culture plates and identified by morphological characterization and matrix-assisted laser desorption/ionization-time of flight mass spectrometry (MALDI-TOF MS).

### Histological preparation of the middle and inner ears

Both wild-type mice (*n* = 4) and Galnt2^tm1Lat/tm1Lat^ mutant mice (*n* = 4) were killed in a time span ranging from 7 days to 9 months. Bullae were fixed in paraformaldehyde (PFA) with 4% concentration at 4°C for 24 h and then in ethylenediaminetetraacetic acid (EDTA) with 10% concentration for decalcification. After dehydration and cleaning, samples were embedded in a paraffin block and cut into 5-μm sections by a rotary microtome (American Optical, Buffalo, New York, USA) to place on slides (Fisher Scientific Co, Ltd., Pittsburgh, PA, USA). Samples were embedded on Tissue-Tek OCT (Sakura Finetek, Torrance, CA, USA) without dehydration and cut into 10-μm sections with a cryostat microtome (Leica, Nussloch, Germany).

### Sample staining

The protocol from Rosen's Lab was used for hematoxylin and eosin (H&E) staining (http://www.bcm.edu/rosenlab), and Mayer's Mucicarmine Staining was used for the identification of goblet cells in accordance with the protocol from Electron Microscopy Sciences. All sample sections were screened under a light microscope (Leica DFC500, Germany).

### Scoring for pathology

A 4-point scoring scale was designed to evaluate the pathology of the middle and inner ear samples, as described in an earlier study (13). For recording the absence of pathology in the middle and inner ears, several symbols were assigned: +, scarce pathology; ++, prevalent pathology; and +++, pathology involving the entire middle and inner ears. Pathologies scored as aforementioned included middle ear effusion, inner ear effusion, infiltration of inflammatory cells, the proliferation of epithelial cells, and density of goblet cells. Category data were measured by the chi-square test.

### Scanning electron microscopy

Wild-type mice (*n* = 3) and Galnt2^tm1Lat/tm1Lat^ mutant mice (*n* = 3) were killed at the ages of 3 months and 6 months. Bullae were dissected and immersed in glutaraldehyde with a 2.5% concentration of cacodylic acid in phosphate-buffered saline (PBS) with 0.1 mol/L (pH = 7.2) at 4°C for one night. Dissection was performed to expose the middle ear cavity. Samples were washed with distilled water 6 times and dehydrated in graded ethanol, after fixing 1% osmium tetroxide in 0.1 M cacodylic acid (pH = 7.2). All samples were dried by the CO_2_ critical point, followed by sputter plumbum coating. All samples were screened by a high-resolution electron microscope (Helios NanoLab 650; FEI, Hillsboro, OR, USA).

### Reverse transcription-polymerase chain reaction analysis

The mice (*n* = 3 in each group) were killed at 7 months, from which bullae were isolated for RNA analysis, immediately as described in the previous section. The primers used for RT-PCR of these mouse genes were laid out as follows:

GAPDH: AACTTTGGCATTGTGGAAGG and GGAGACAA CCTGGTCCTCAG;

TNF-α: CCACCACGCTCTTCTGTCTAC and CCTTGAAGA GAACCTGGGAGT;

TGF-β1: AGCCCGAAGCGGACTACTAT and TCCACATGTTG CTCCACACT;

Muc5a: TGGAAGGATGCTATCCCAAG and CACCAGCAT TGTGGGTACAG;

Muc5b: GACACCATCTATGGGGTTGG and CAGGACTGTT CACCCAGGTT;

The PCR products were subjected to agarose gel electrophoresis in which the intensity of the gray band was digitalized and calculated using the ImageJ software and referred by GAPDH.

### Immunofluorescent staining for TNF-α and TGF-β and proliferating cell nuclear antigen

All sections were washed with PFA at 1.5% concentration for 10 min and then with PBS two times for 5 min. Next, the sections were permeabilized in Triton X-100 with 0.5% concentration for 5 min, then washed with PBS two times for 5 min, and finally blocked in goat serum of 3% concentration + bovine serum albumin (BSA) of 2% concentration for 1 h. All samples were immersed in anti-TNF-α, anti-TGF-β, or anti-PCNA (1:200 dilution) subsequently and incubated at 4°C for one night. All samples were immersed in the antibody Alexa 488 (1:400 dilution) again for 1 h after washing two times with PBS for 5 min. The 4′,6-diamidino-2-phenylindole (DAPI) staining of samples was also performed for 15 min. Finally, Vectashield antifade mounting medium (Vector Laboratories, Burlingame, CA) was used for preserving the fluorescence of the section, and an immunofluorescence microscope (DFC500, Leica Co, Ltd., Germany) was used for further observation.

## Results

### Characteristics of *Galnt2*

*Galnt2* encodes a member of the glycosyltransferase 2 protein family. It is involved in the o-linked glycosylation *via* serine and threonine. The galnt2 genome DNA contains 10 exons. The *Galnt2*(-/-) mutant used in this study was the deletion of exon 7 that contained the amino acid residues DSHCEC crucial for GALNT2 catalytic activity (Figure 1A). The present study designed primers near the exon 7 position, identified the wild type galnt2 and mutant galnt2 by PCR, and verified the successful construction of galnt2 mutants (Figure 1B).

### Gross observation of the middle ear

**Figure 2:**
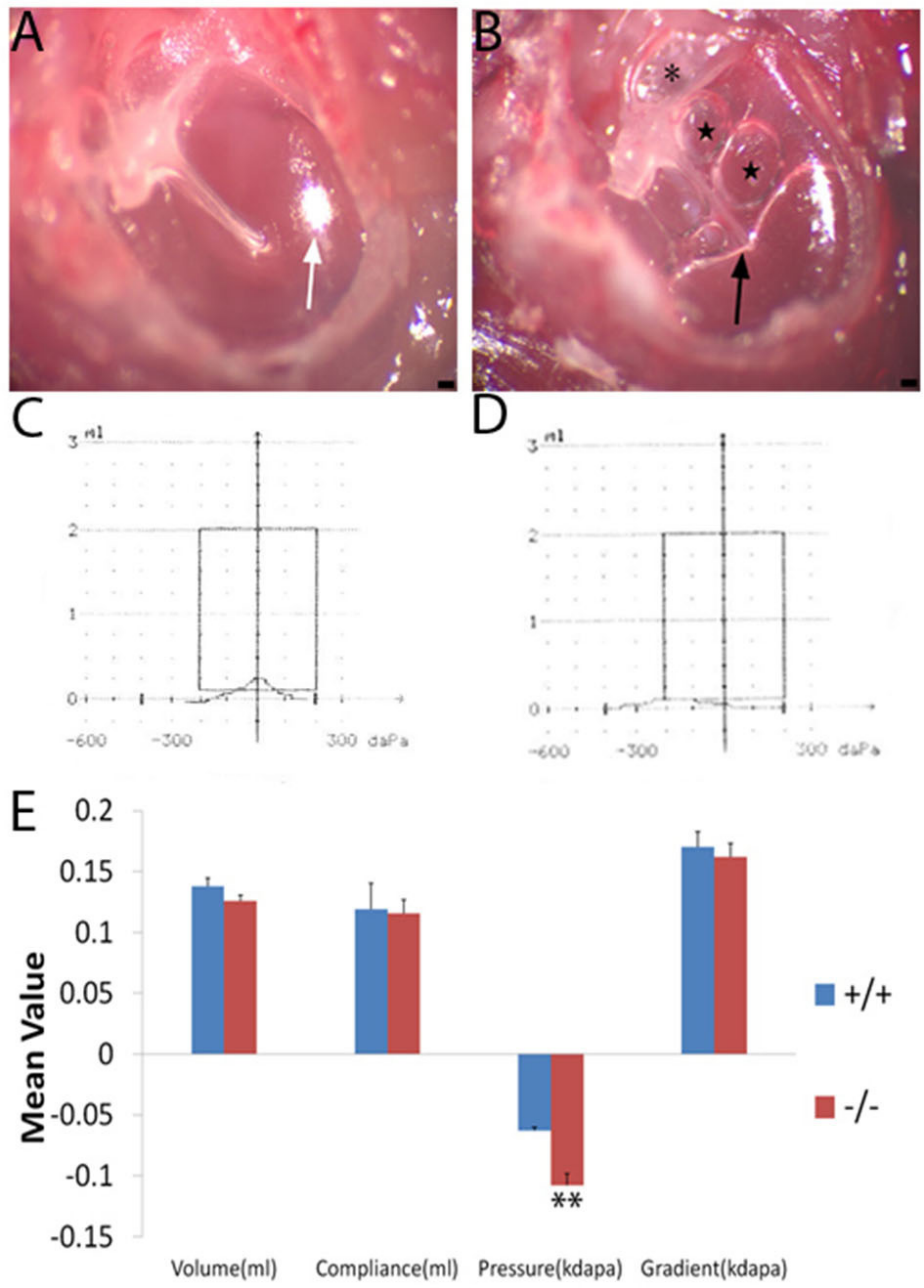
Tympanic membrane image and a comparison of the mean value of tympanometry parameters. Nine wild-type mice (+/+) and eight mutant mice (-/-) were used to measure the tympanometry at the age of 2 months. **(A, B)**: Tympanic membrane image (*n* = 3, each group) under an anatomical microscope. The wild-type mouse **(A)** displays a normal tympanic membrane with a subuliform light cone (white arrow) on its anteroinferior quadrant. In the ear of the mutant mouse **(B)**, the retraction and adherence of the tympanal flaccid part (asterisk) were visible, hyperemia around the malleus, effusion (black arrow), and bubbles (star) in the middle ear cavity and an abnormal light cone were detected. **(C, D)**: The wild-type **(C)** mouse shows a normal A-type curve with a peak at 0 pressure. The mutant mouse **(D)** exhibits an abnormal C-type curve with a peak at negative pressure. **(E)** Significant differences in pressure were noticed between the wild type and mutant mice at the age of 2 months. Error bar represents the standard error (SE) of the mean. *P < 0.05, **P < 0.01. Scale bar = 50 μm.

The tympanic membrane of the wild-type and the mutant (Figure 1) mouse at 2 months of age was, respectively, observed under an anatomical microscope. The mutant mice exhibited severe effusion of the middle ear cavity, retraction and adherence of the tympanal flaccid part, hyperemia around the melleus, and light-cone deformation and disappearance, when compared with the wildtype ones (Figures 2A, B). The effusion of the middle ear cavity and the retraction and adherence of the tympanal flaccid part were characteristic manifestations of the OM.

### The function of the middle ear by the tympanometry assessment

Wild-type (*n* = 9) and mutant mice (*n* = 8) at 2 months of age were measured by tympanometry to assess the function of the middle ear. The tympanogram results were presented for comparison (Figure 2E). There was no difference in volume between wild-type and mutant mice. Similarly, for the compliance and gradient, no difference was found between the wild-type mice and the mutant ones. However, the negative pressure of the middle ear was more significant in the mutant mice when compared with the wild-type ones. Negative pressure was correlated with otitis media and was more likely to cause effusion in the middle ear cavity (14, 15). The tympanogram of a 2-month-old mouse in the wild-type group represented the normal curve (Figure 2C), while that in the mutant group represented the abnormal curve (Figure 2D).

### ABR thresholds and DPOAE

**Figure 3:**
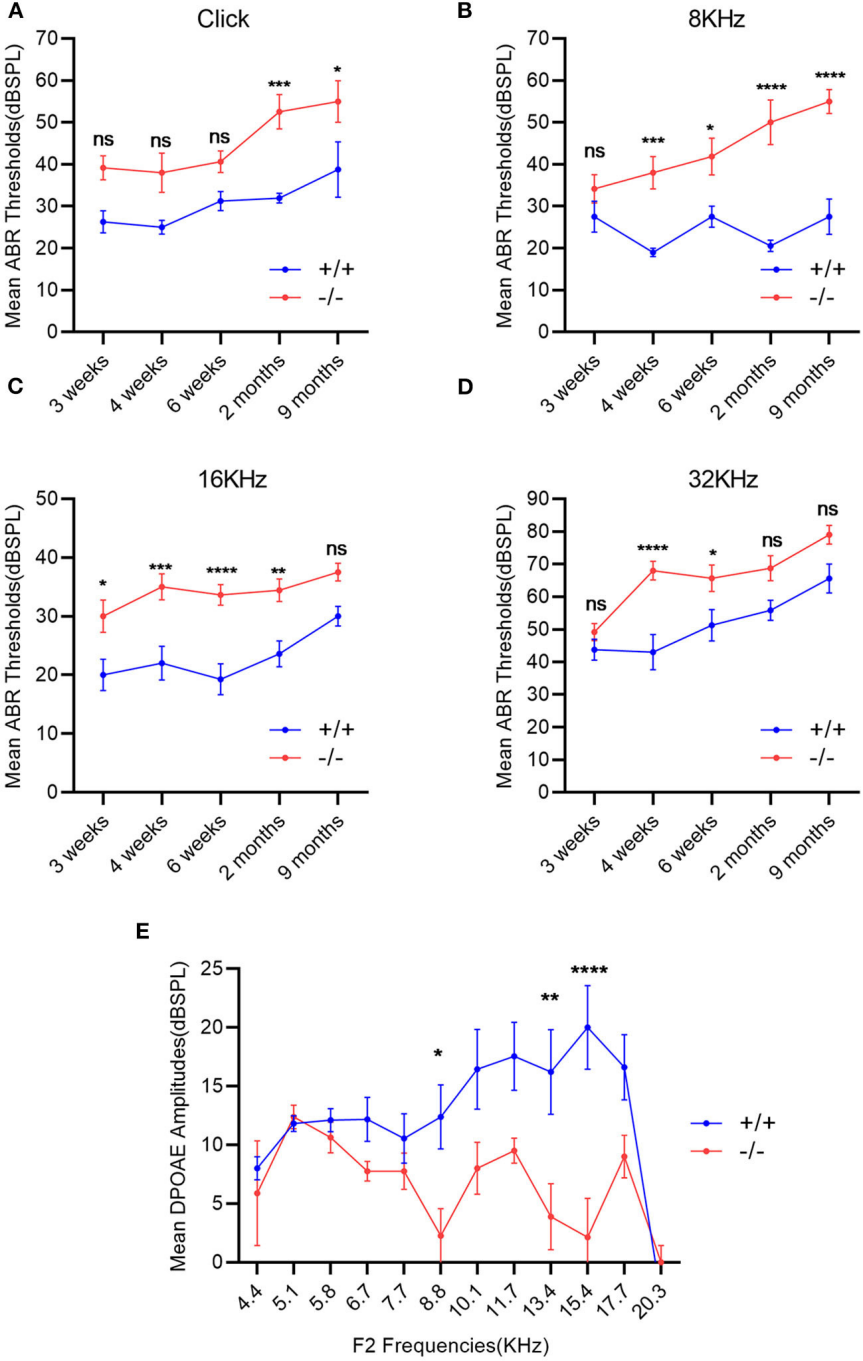
Evaluation of hearing impairment through auditory brainstem response (ABR) and distortion product otoacoustic emission (DPOAE). **(A–D)**: Wild-type mice (+/+, *n* = 14) and mutant mice (-/-, *n* = 14) were measured using the ABR thresholds at different stimulus frequencies and different time points. The mutant mice showed significantly higher mean ABR thresholds at each stimulus frequency and at several time points compared with the wild-type mice. **(E)** DPOAE amplitudes were recorded in wild-type mice (+/+, *n* = 9) and mutant mice (-/-, *n* = 8) at the age of 2 months. The mean DPOAE amplitudes of mutant mice were significantly lower than those of the wild-type mice at 8.8 KHz, 13.4 and 15.4 KHz. The error bar represents the standard error (SE) of the mean. *P < 0.05, **P < 0.01, ***P < 0.001, ****P < 0.0001.

The ABR thresholds of mice from 3 weeks to 9 months were consistently measured in both groups. The mean ABR thresholds were 55 (click stimuli), 40 (8 KHz), 35(16 KHz), and 60 (32 KHz) decibel of sound pressure level (dBSPL), respectively, indicating the hearing impairment (16). Along with the growth of age, the ABR thresholds increased in both wild-type and mutant mice groups, especially in the mutant ones. The mean ABR thresholds of mutant mice were significantly higher than those of the wild-type ones at each stimulus frequency and at several time points compared with the wild-type mice (Figures 3A–D). The mean DPOAE amplitudes of mutant mice were markedly lower when compared with those of the wild-type mice at frequencies of 8.8, 13.4, and 15.4 KHz (Figure 3E).

### Abnormality in mutant mice by measurement of the Eustachian tube

**Figure 4:**
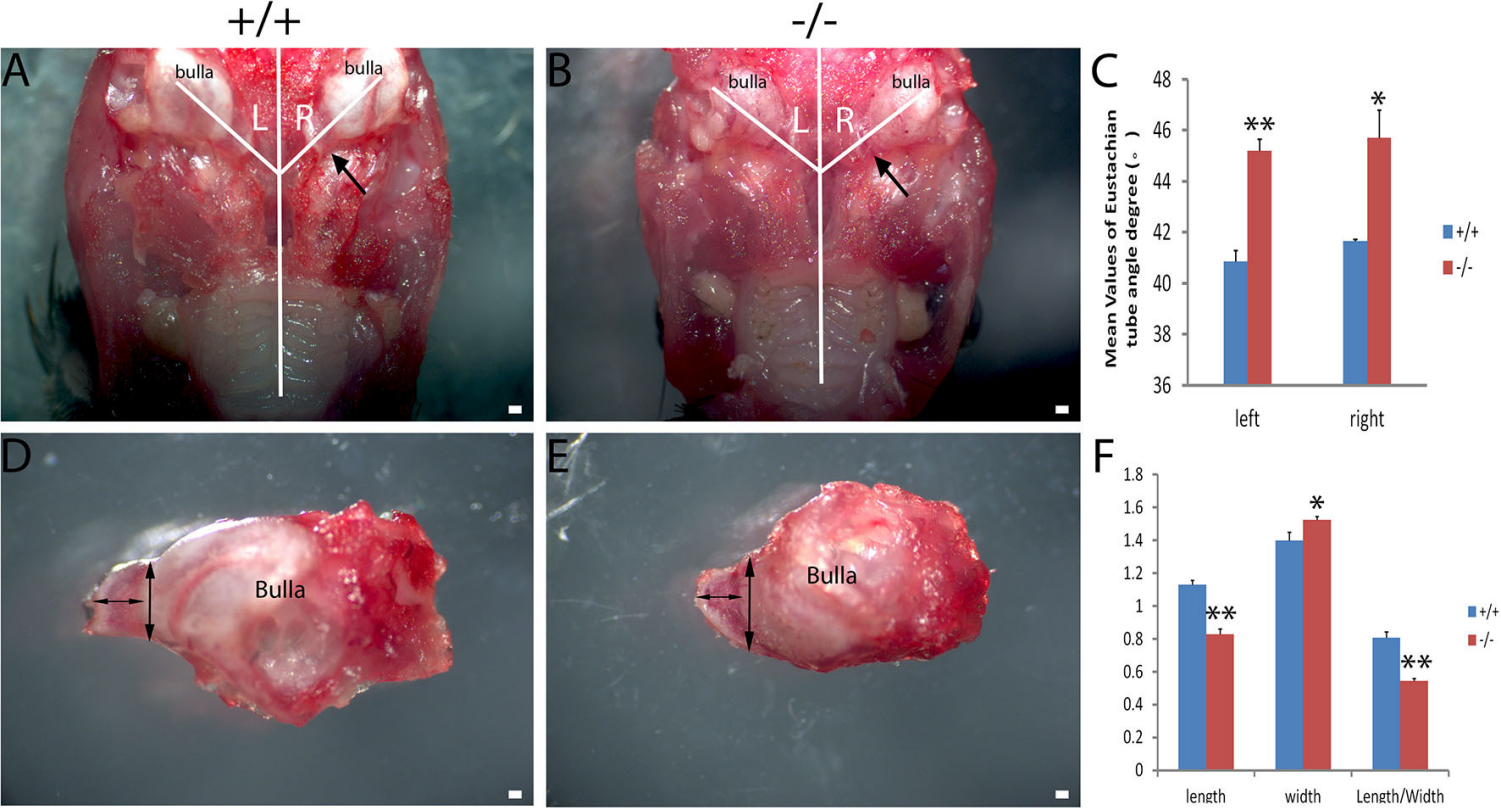
Assessment of the Eustachian tube's dysmorphology in Galnt2^tm1Lat/tm1Lat^ mice. Wild-type (+/+) and mutant mice (-/-) at the age of 2 months (*n* = 6 for each genotype, equal numbers of male and female mice were selected) were used to measure the intersection angle between the midline and the Eustachian tube **(A, B)** and the length and width of the bony part of the Eustachian tube **(D, E)**. **(C)** The intersection angle is significantly larger in mutant mice, compared with the wild-type mice. Wild-type and mutant mice were used to measure the length and width of the bony part of the Eustachian tube **(D, E)**. **(F)** The mean length of the Eustachian tube in wild-type mice is longer than that of the mutant mice, whereas the mean width of the Eustachian tube in mutant mice is wider compared with the wild-type mice. The length/width ratio in wild-type mice is greater than that of the mutant mice. The arrow shows the location of the Eustachian tube. The double-ended arrow shows the length and width of the bony part of the Eustachian tube. The error bar represents the standard error (SE) of the mean. *P < 0.05, **P < 0.01. Scale bar = 200 μm.

The craniofacial abnormalities increased the susceptibility to otitis media (17, 18) which were measured between the wild-type and mutant groups matched by age. The 2-month-old wild type (+/+) and the mutant mice (-/-) (*n* = 6 in each genotype, including three male and three female mice) were used to measure the intersection angle between the midline and the Eustachian tube (Figures 4A, B). The intersection angle of the mutant mice was significantly larger than that of the wild-type mice (Figure 4C). The length and the width of the bony component of the Eustachian tube (Figures 4D, E) were also measured. A longer mean length of the Eustachian tube of wild-type mice was observed when compared with the mutant mice, whereas there was an increase in the mean width of the Eustachian tube of mutant mice when compared with the wild type mice. The length/width ratio of the wild type mice was higher than that of the mutant ones (Figure 4F). The Eustachian tube of mutant mice was shorter, wider, and more horizontal, which may increase the predisposition to OM.

### Isolation of pathogenic bacteria from the middle ears of the mutant mice

Bacteria and their secretion are acknowledged as the most common causes of OM with effusion (19). To investigate the causative agent of OM in mutant mice, lavage of the middle ear from both types of 3-month-old mice was performed. *Bordetella hinzii* was specifically isolated from the middle ears of the mutant mice, whereas nothing was isolated from the middle ears of the wild-type mice. *Bordetella hinzii* belongs to the *Bordetella* genus, which was reported as a Gram-negative and short rod-shaped bacterium with positive oxidase and catalase isolated from both humans and mice suffering from cystic fibrosis (20).

### Histopathologic evidence of OM in the mutant mice

**Figure 5:**
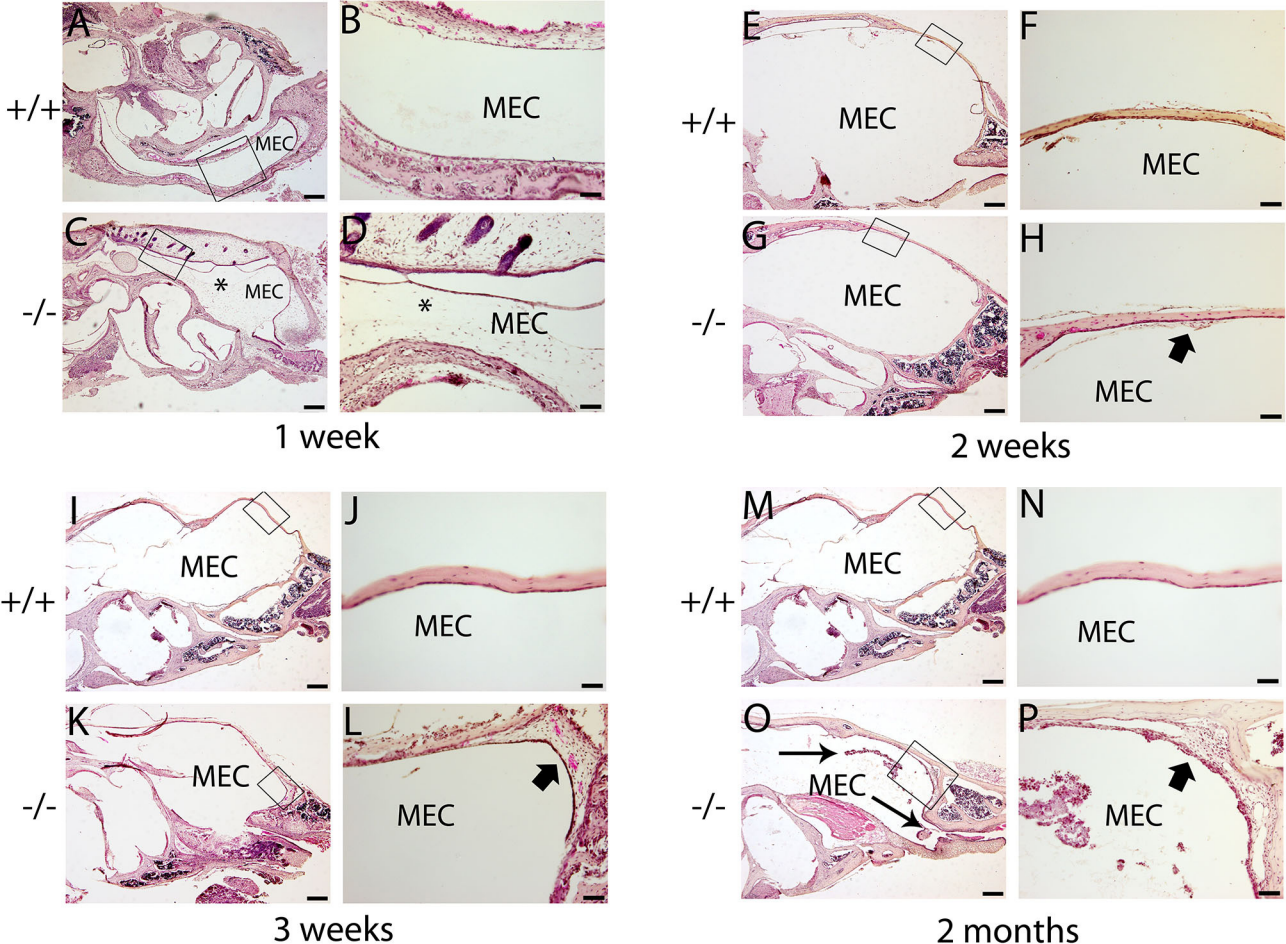
Hematoxylin and eosin (H&E) histology shows the development of middle ear structures and pathology. Wild type (+/+) and mutant mice (-/-) were observed for the morphology of the middle ear at each time point (*n* = 4 for each genotype). In each row, the right panel represents the enlarged image corresponding to the rectangular region on the left. **(A–D)** Representative images of mice at the age of 1 week. The middle ear cavity (MEC) was filled with mesenchymal cells [asterisk, **(C, D)**] in mutant mice, whereas the middle ear cavity was large and clean in wild-type mice **(A, B)**. **(E–H)**: Representative images of mice at the age of 2 weeks. The mesenchymal cells disappeared in mutant mice, and the middle ear cavity was clean in both groups **(E, G)**. The mucosa of the middle ear cavity becomes thicker (short arrow) in mutant mice compared with the monolayer epithelium in wild-type mice **(F, H)**. **(I–L)**: Representative images of mice at the age of 3 weeks. There was no obvious effusion in mutant mice, compared with the wild-type mice **(I, K)**. The mucosa and submucosa hyperplasia (short arrow) developed further and capillary proliferation occurred in the mutant mice **(L)**. **(M–P)**: Representative images of mice at the age of 2 months. In wild-type mice, neither effusion nor inflammation was detected, and the middle ear cavity was covered with monolayer epithelium **(M, N)**. In mutant mice, inflammatory infiltration and debris pervaded the middle ear cavity (long arrow), the mucosa and submucosa hyperplasia progressed to a much greater severity (short arrow), and as regards the capillary proliferation and expansion, the Eustachian tube exhibited general thickening with the proliferation of epithelial cells **(O, P)**. Scale bars: 200 μm **(A, C, E, G, I, K, M, O)**; 50 μm **(B, D, F, H, J, L, N, P)**. MEC, middle ear cavity.

**Figure 6:**
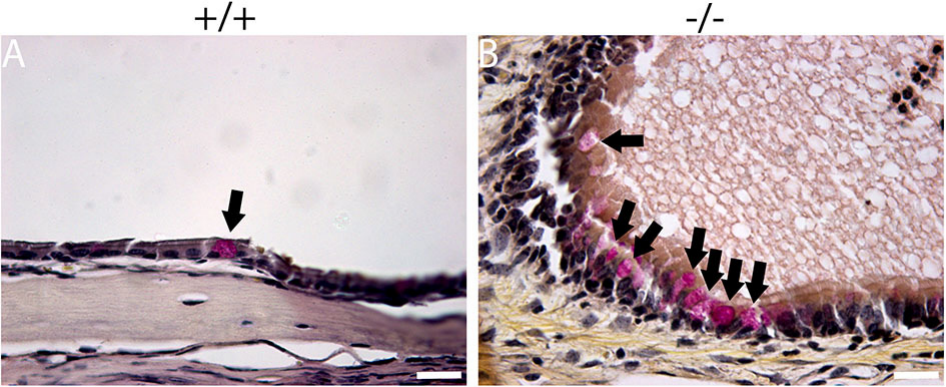
Mucin staining reveals goblet cells in mutant mice. Representative sections show the presence of goblet cells (arrow) in the mucosa of the middle ear cavity of wild-type and mutant mice at the age of 2 months. **(A)** Goblet cells were found to be sparsely scattered among other cells in the mucosa of wild-type mice. **(B)** By contrast, goblet cells were present at high density among other cells in the mucosa of mutant mice. Scale bars: 20 μm.

The histopathology of the middle ear was evaluated to track the OM progression at different ages. In 1-week-old mice, the middle ear cavity was filled with mesenchymal cells in mutant mice, whereas the middle ear cavity was large and clean in wild-type mice (Figures 5A–D). At the age of 2 weeks, the mesenchymal cells disappeared in mutant mice and the middle ear cavity was clean in both groups. The mucosa of the middle ear cavity became thicker in mutant mice than in wild-type mice (Figures 5E–H). In 3-week-old mice, no obvious effusion occurred in mutant mice compared with the wild-type mice. However, the mucosa and submucosa hyperplasia developed further, and capillary proliferation occurred in the mutant mice (Figures 5I–L). At the age of 2 months, in wild-type mice, neither effusion nor inflammation was detected, and the middle ear cavity was covered with monolayer epithelium. In mutant mice, the middle ear cavity was filled with inflammatory infiltration and debris; the mucosa and submucosa hyperplasia progressed to a much greater severity; as regards the capillary proliferation and expansion, the Eustachian tube exhibited general thickening with the proliferation of epithelial cells (Figures 5M–P). Sparsely scattered Goblet cells were found in the mucosa of wild-type mice (Figure 6A). By contrast, goblet cells were presented with high density in the mucosa of mutant mice (Figure 6B). To assess the pathology in OM, a semi-quantitative evaluation was performed (Table 1). It suggested that the mutant mice exhibited an onset of the middle ear pathology at the age of 2 weeks and progressed to typical OM at the age of 2 months.

**Table 1:** Semi-quantitative evaluation of the middle ear pathology in mutant mice.

**Mouse ID**	**Genotype**	**Age**	**Effusion**	**Inflammatory cells**	**Tissue debris**	**Tissue hyperplasia**	**Goblet cells**
1	+/+	2W	-	-	-	-	-
2	+/+	2W	-	-	-	-	-
3	+/+	2W	-	-	-	-	-
4	+/+	2W	-	-	-	-	-
5	+/+	3W	-	-	-	-	+
6	+/+	3W	-	-	-	-	-
7	+/+	3W	-	-	-	+	-
8	+/+	3W	-	-	-	-	-
9	+/+	2M	-	-	-	+	+
10	+/+	2M	-	-	-	-	-
11	+/+	2M	-	-	-	-	-
12	+/+	2M	-	-	-	-	+
13	-/-	2W	-	-	-	+	-
14	-/-	2W	-	-	-	+	-
15	-/-	2W	-	-	-	+	+
16	-/-	2W	-	-	-	+	+
17	-/-	3W	-	+	+	++	+
18	-/-	3W	-	-	-	++	++
19	-/-	3W	+	-	-	++	+
20	-/-	3W	-	-	-	++	++
21	-/-	2M	+++	++	++	++	++
22	-/-	2M	+++	+++	+++	+++	+++
23	-/-	2M	+++	+++	+++	+++	+++
24	-/-	2M	+++	+++	+++	+++	+++

To exhibit the degree of pathological alteration in the mutant mice, symbols (-, +, ++, +++) were used, with (-) indicating no pathology and (+) to (+++) indicating a mild-to-severe degree of pathology, respectively. The criteria for pathology included middle ear effusion, inflammatory cells, tissue debris, tissue proliferation, and density of goblet cells. The maximum possible score per mouse was 15 points (1 point for each +). The total score rate (84/180) of the mutant mice was significantly higher than that (4/180) of the wild-type mice (P < 0.01, χ2 test).

### Cilia impairment in mutant mice by SEM

**Figure 7:**
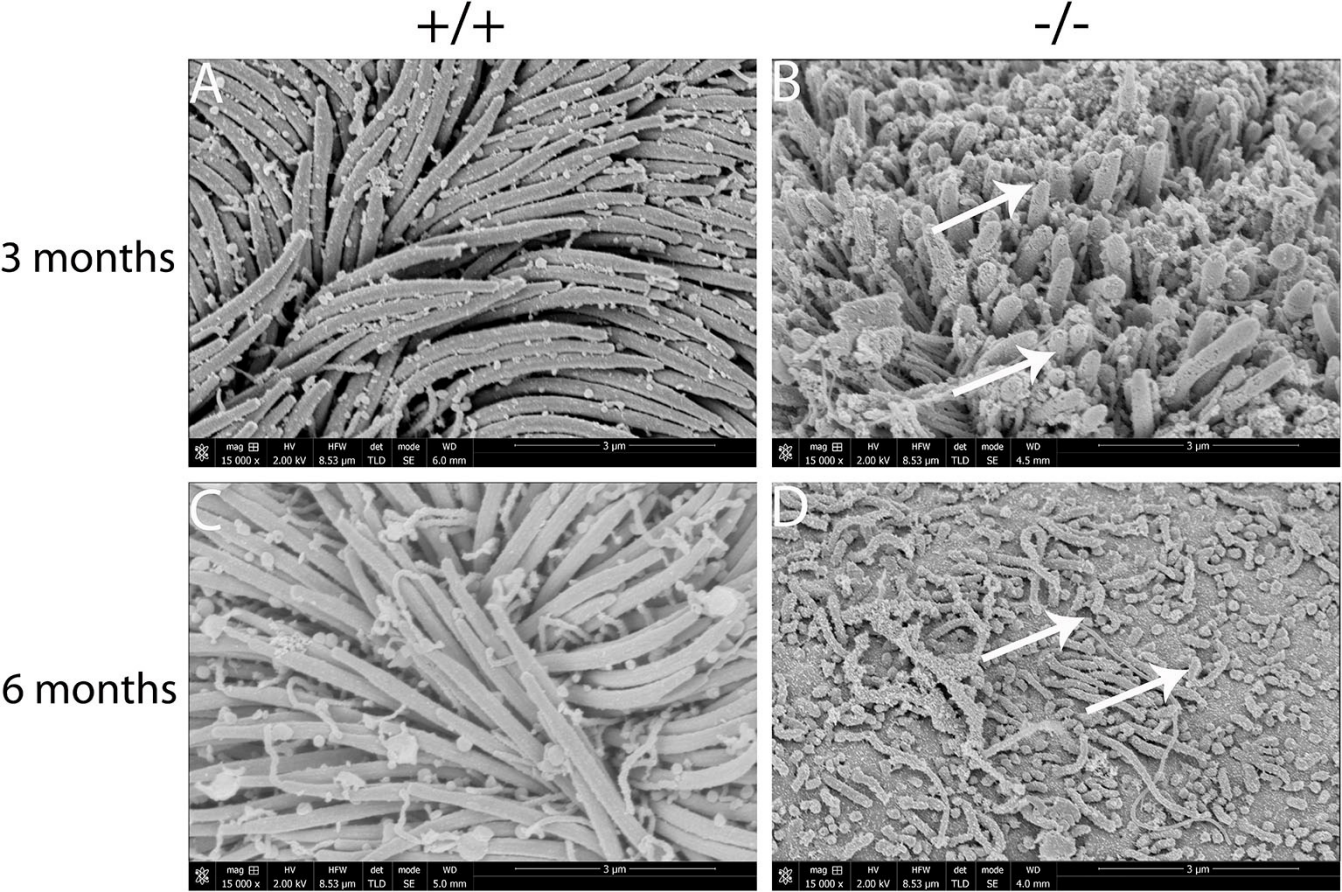
Scanning electron microscopy (SEM) observation of the cilia of the middle ear cavity in mutant mice. Wild-type (+/+) and mutant mice (-/-) were observed in the SEM of the middle ear at the ages of 3 and 6 months, respectively (*n* = 3 for each genotype). Wild-type mice exhibited a thick lawn of morphologically normal and distributed cilia in the mucosa of the middle ear at both time points **(A, C)**. The cilia of the mucosa of the middle ear in mutant mice were short, impaired, and disrupted (arrows), and the impairment of the cilia progressed to a much greater severity at 6 months than the cilia at 3 months **(B, D)**. Scale bars: 3 μm.

The present study assessed the cilia impairment of mucosa in mutant mice at two time points through a scanning electron microscope. A thick lawn of morphologically normal and distributed cilia was observed in the mucosa of the middle ear at both of the time points. For mutant mice, the short cilia of mucosa in the middle ear were observed with impairment and disruption. The impairment of the cilia was more serious at 6 months than at 3 months (Figures 7A–D). These results suggest that the mutant mice have difficulty in draining the secretion in the middle ear cavity, which may lead to OM.

### Upregulated inflammatory cytokine in mutant mice by RT-PCR and immunofluorescence

**Figure 8:**
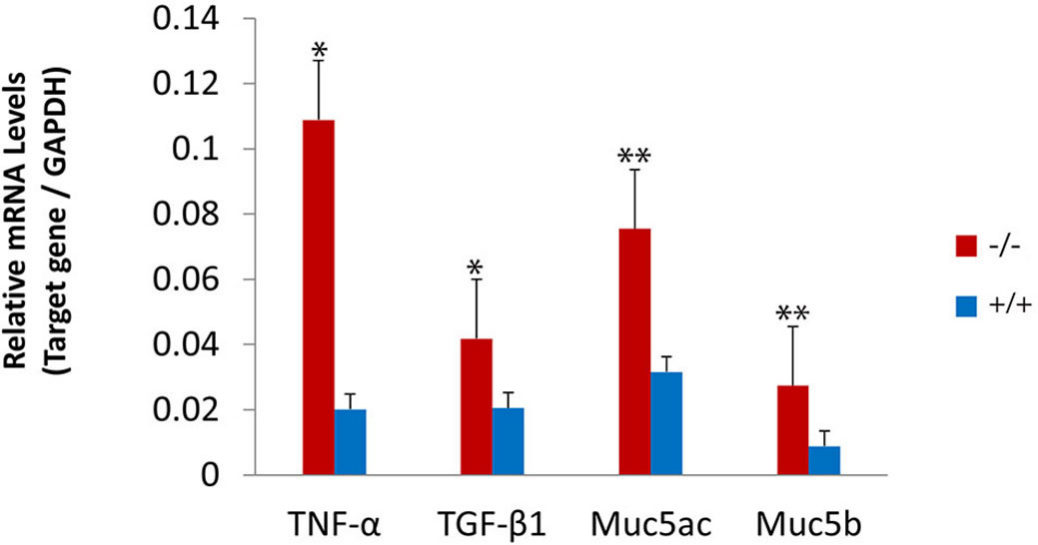
Upregulated expression of the inflammatory-related gene in mutant mice. The levels of gene expression were evaluated in wild-type (*n* = 3) and mutant mice (*n* = 3) at the age of 7 months. The messenger RNA (mRNA) accumulation levels of tumor necrosis factor-alpha (TNF-α), transforming growth factor-beta 1 (TGF-β1), Muc5ac, and Muc5b were significantly higher in mutant mice than in wild-type mice. The error bar represents the standard error (SE) of the mean. *P < 0.05, **P < 0.01.

**Figure 9:**
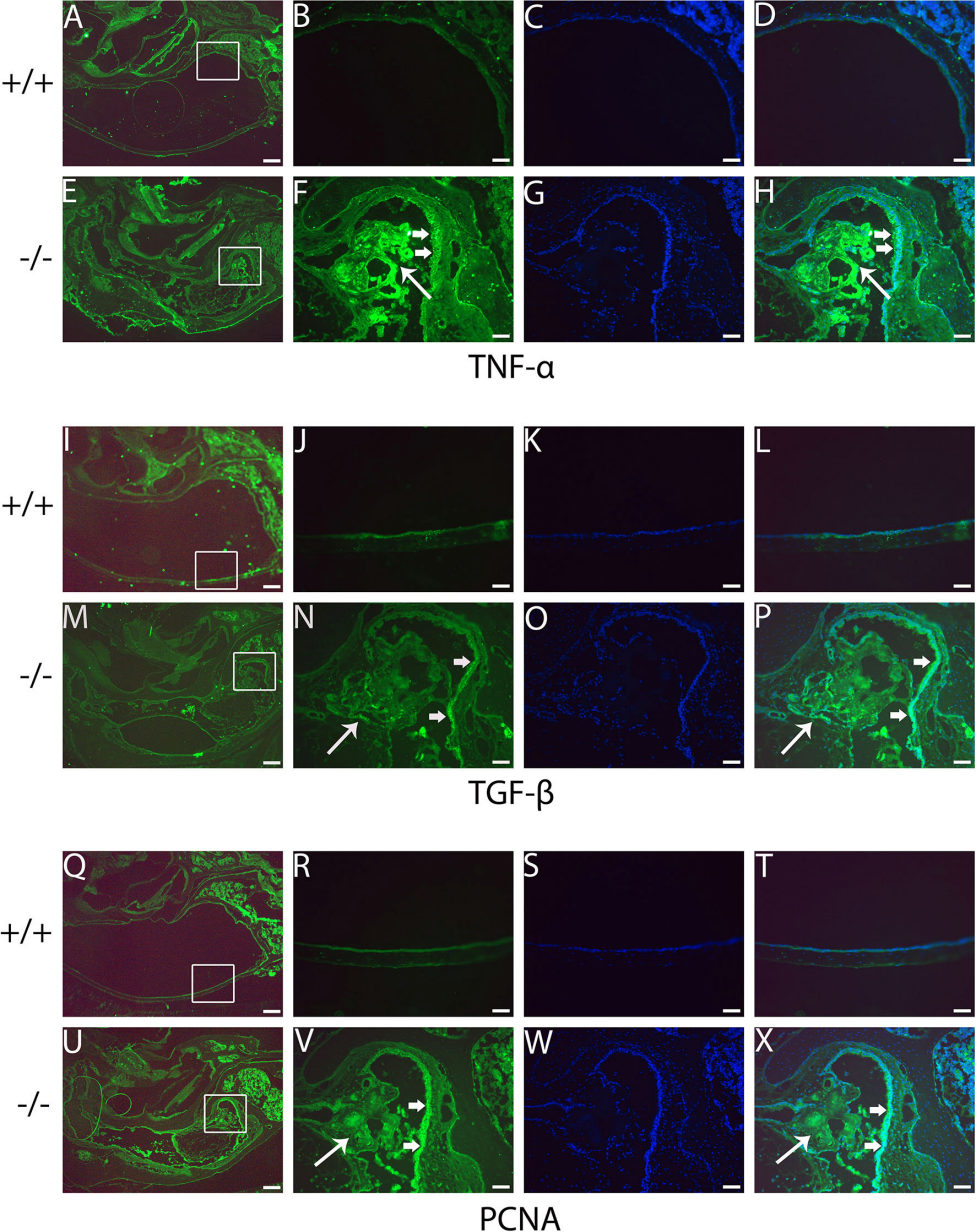
Immunofluorescent detection of pro-inflammatory proteins such as tumor necrosis factor-alpha (TNF-α), transforming growth factor-beta (TGF-β), and proliferating cell nuclear antigen (PCNA) in mutant mice. Representative images from 6-month-old wild type (+/+) and mutant (-/-) mice (*n* = 3 for each group) stained with anti-TNF-α, anti-TGF-β, and anti-PCNA antibody and revealed with Alexa Fluor 488 (green). **(A, E, I, M, Q, U)** show gross morphology of the middle ear. **(B, F, J, N, R, V)** represent the enlarged image corresponding to the rectangular region in the left. Images **(C, G, K, O, S, W)** counterstained with 4′,6-diamidino-2-phenylindole (DAPI) reveal the nuclei in these tissues. **(D, H, L, P, T, X)** on the right are merged with the images **(B, F, J, N, R, V)** and the images were counterstained with DAPI. The intensities of TNF-α, TGF-β, and PCNA are all stronger in the mutant mice than in control mice **(F, N, V, H, P, X)**. The expression of TNF-α in the inflammatory tissue of the middle ear cavity (short arrow) is higher than that in the mucosa of the middle ear cavity (long arrow) **(F, H)**. The expression of TGF-β and PCNA is higher in the mucosa of the middle ear cavity (short arrow) compared to the inflammatory tissue of the middle ear cavity (long arrow) **(N, V, P, X)**. Scale bars: 200 μm **(A, E, I, M, Q, U)**; 50 μm **(B–H, J–L, N–P, R–T, V–X)**.

The present study assessed the expression of the inflammation-related gene in mutant mice at the age of 7 months. RT-PCR revealed that the messenger RNA (mRNA) of TNF-α, transforming growth factor beta 1 (TGF-β1), Muc5ac, and Muc5b were significantly higher in mutant mice than in wild-type mice (Figure 8). To evaluate the protein levels of TNF-α and TGF-β1, sections of the middle ear of wild-type and mutant mice at the age of 6 months were stained with anti-TNF-α (Figures 9A–H), anti-TGF-β (Figures 9I–P), and anti-PCNA (Figures 9Q–X) antibody. The intensity of TNF-α, TGF-β, and PCNA was all higher in the mutant mice than in control (Figures 9F, N, V, H, P, X). The expression of TNF-α in the inflammatory tissue of the middle ear cavity was higher than that in the mucosa of the middle ear cavity (Figures 9F, H). The expression of TGF-β and PCNA was higher in the mucosa of the middle ear cavity compared to the inflammatory tissue of the middle ear cavity (Figures 9N, V, P, X).

## Discussion

In this study, we have proven that *Galnt2* mutant mice were prone to OM. The *Galnt2* mutant OM mouse model was similar to the one with the *Hyp-Duk/Y* mutant, which exhibited pus and effusion in the middle ear cavity (5). The OM of mutant mice occurred at the age of 2 weeks. The mutant mice harboring otitis media were characterized by effusion and capillary expansion. Increased goblet cells and PCNA staining indicated mucosa proliferation (21, 22). Effusion originates from the infection and inflammation of the middle ear and is exacerbated by dysfunction of the Eustachian tube and mucociliary impairment (23).

The Eustachian tube is coated with pseudostratified ciliary columnar epithelium and plays an important role in ventilation and secretion clearance (24). Bacteria resident in the upper respiratory tract are able to retrograde infection through the Eustachian tube when its function of ventilation and clearance is poor (16). Eustachian tube with horizontal orientation may impair the function of ventilation and clearance, since the normal position and morphology of Eustachian tube have protective function of the middle ear against otitis media (25). As in the setting of the *Galnt2* mutant mice, the Eustachian tube developed to wide and short shape, with an increased intersection angle between the midline and the Eustachian tube, making the Eustachian tube locate to a more horizontal position. Both malformation and dysfunction of the Eustachian tube interfering with ventilation and clearance of middle ear may result in OM.

Hearing loss was found in the *Galnt2* mutant mice from 3 weeks old. The tympanograms showed that the negative pressure of the middle ear was more evident in mutant mice, compared to the wild type mice. The ABR thresholds of the mutant mice were significantly higher when compared with those of wild type ones. The DPOAE confirmed lower amplitudes in mutant mice. The mucosa of middle ear cavity was thicker at the age of 2 weeks and gradually developed to cause inflammatory infiltration, mucosa proliferation, and capillary expansion at the age of 2 months. PCNA, an active cell proliferation marker, was highly associated with otitis media (Lim et al., 1971). Our findings depicted that the intensity of PCNA was higher in the mutant mice than in control ones, in accordance with the pathology of the middle ear. Goblet cells in the middle ear mucosa are one of the mucosal secretions responsible for the immune system (26). A high density of goblet cells in the mucosa of mutant mice was seen, which might result in an excess effusion in the middle ear cavity. Cilia in the middle ear are critical for the clearance of mucosal secretions, while the abnormal cilia impair normal mucociliary clearance and exacerbate clinical complications (27). Short cilia with impairment and disruption of mucosa in the middle ear were observed and tended to be more serious with the increasing age of mutant mice, revealing that the impairment of cilia elicited significant function in the occurrence of OM.

Inflammation increased with gene levels of TNF-α, TGF-β1, Muc5ac, and muc5b in the middle ear in mutant mice, which were consistent with previous human and animal studies (28). Consistent with our initial hypothesis, the TNF-α levels were significantly upregulated by mutating GALNT2. TNF-α triggers the transcription factor, such as nuclear factor-kappa B (NF-κB), as the basis of various physiological and pathological procedures (29). The *Bordetella hinzii* infection in the middle ear cavity of *Galnt2* mutant mice contributes to inflammation, which upregulates TNF-α and promotes further pathological processes. TGF-β1 controls cellular proliferation and differentiation, and epithelial-mesenchymal transformation, since the TGF-β signaling pathway plays an important role both in tooth and craniofacial development (30). Additionally, the TGF-β1 upregulation in *Galnt2* mutant mice may play a role in the development of the Eustachian tube. Mucins are responsible for the gel-like characteristics of mucoid middle ear fluids. Both Muc5a and Muc5b were found to determine the properties of airway mucus gel (31). The upregulation of Muc5b in the ear may have a role in middle ear effusions (28). These proteins protect the mucosa of the middle ear from pathogen invasion and contribute to the clearance of pathogens. Moreover, upregulation of Muc5ac and Muc5b in *Galnt2* mutant mice resulted in increased middle ear effusion and decreased mucociliary clearance, thereby preventing otitis media.

Admittedly, several limitations should be acknowledged in the present study. First, this is an animal study that includes the uncertainty regarding how truly it is reflected in human studies. Second, future exploration of OM's pathological features induced by Galnt2 mutation is essential to reveal the underlying mechanisms.

## Conclusion

To our knowledge, the current study was the first to report on the occurrence of OM in the Galnt2 ^tm1Lat/tm1Lat^ mutant mice. The present study suggests that the mutant *Galnt2* gene may result in the inflammation of the middle ear and mimic human OM. This study suggests the reason that OM in *Galnt2* mutant mice is highly associated with hearing loss, which is mainly presented as a dysfunction of the Eustachian tube, mucosa proliferation, and capillary expansion.

## Data availability statement

The original contributions presented in the study are included in the article/supplementary material, further inquiries can be directed to the corresponding authors.

## Ethics statement

The animal study was reviewed and approved by Health Sciences Institutional of Animal Care Center and Ethics Committee of Case Western Reserve University.

## Author contributions

MX and YC contributed to the conception and design of the study. HLi, JH, and YG contributed to the acquisition of data. HLv and WM performed the experiments. XZ and QZ contributed to the analysis of data. WM wrote the manuscript. MX revised the manuscript. All authors reviewed and approved the final version of the manuscript.

## References

[1] Sundgaard JV Harte J Bray P Laugesen S Kamide Y Tanaka C. Deep metric learning for otitis media classification. Med Image Anal. (2021) 71:102034. 10.1016/j.media.2021.102034 33848961

[2] Monasta L Ronfani L Marchetti F Montico M Brumatti LV Bavcar A. Burden of disease caused by otitis media: systematic review and global estimates. PLoS ONE. (2012) 7:e36226. 10.1371/journal.pone.0036226 22558393 PMC3340347

[3] Vanneste P Page C. Otitis media with effusion in children: pathophysiology, diagnosis, and treatment. A review. J Otol. (2019) 14:33–9. 10.1016/j.joto.2019.01.005 31223299 PMC6570640

[4] Darrow DH Dash N Derkay SC. Otitis media: concepts and controversies. Curr Opin Otolaryngol Head Neck Surg. (2003) 11:416–23. 10.1097/00020840-200312000-00002 14631172

[5] Han F Yu H Li P Zhang J Tian C Li H. Mutation in Phex gene predisposes BALB/c-Phex(Hyp-Duk)/Y mice to otitis media. PLoS ONE. (2012) 7:e43010. 10.1371/journal.pone.0043010 23028440 PMC3461009

[6] Davidoss N Varsak Y Santa Maria. P. Animal models of acute otitis media–a review with practical implications for laboratory research. Eur Ann Otorhinolaryngol Head Neck Dis. (2018) 135:183–90. 10.1016/j.anorl.2017.06.013 29656888

[7] Willett DN Rezaee RP Billy JM Tighe MB DeMaria TF. Relationship of endotoxin to tumor necrosis factor–α and interleukin-1β in children with otitis media with effusion. Ann Otol Rhinol Laryngol. (1998) 107:28–33. 10.1177/000348949810700106 9439385

[8] Teslovich TM Musunuru K Smith AV Edmondson AC Stylianou IM Koseki M. Biological, clinical and population relevance of 95 loci for blood lipids. Nature. (2010) 466:707–13. 10.1038/nature09270 20686565 PMC3039276

[9] Yang X Ongusaha PP Miles PD Havstad JC Zhang F So WV. Phosphoinositide signalling links O-GlcNAc transferase to insulin resistance. Nature. (2008) 451:964–9. 10.1038/nature06668 18288188

[10] Goth CK Halim A Khetarpal SA Rader DJ Clausen H Schjoldager TBG. A systematic study of modulation of ADAM-mediated ectodomain shedding by site-specific O-glycosylation. Proc Nat Acad Sci. (2015) 112:14623–28. 10.1073/pnas.1511175112 26554003 PMC4664366

[11] Qin Z Wood M Rosowski JJ. Measurement of conductive hearing loss in mice. Hear Res. (2010) 263:93–103. 10.1016/j.heares.2009.10.002 19835942 PMC2866764

[12] Zheng QY Tong YCI Alagramam KN Yu H. Tympanometry assessment of 61 inbred strains of mice. Hear Res. (2007) 231:23–31. 10.1016/j.heares.2007.05.011 17611057 PMC2000814

[13] Han F Yu H Tian C Li S Jacobs MR Benedict-Alderfer C. Role for toll-like receptor 2 in the immune response to *Streptococcus pneumoniae* infection in mouse otitis media. Infect Immun. (2009) 77:3100–8. 10.1128/IAI.00204-09 19414550 PMC2708554

[14] Falk B. Sniff-induced negative middle ear pressure: study of a consecutive series of children with otitis media with effusion. Am J Otolaryngol. (1982) 3:155–62. 10.1016/S0196-0709(82)80048-3 7102952

[15] Kanai R Kaneko K. Negative middle ear pressure and otitis media with effusion after surgery under general anesthesia. Acta Otolaryngol. (2012) 132:1049–53. 10.3109/00016489.2012.687455 22779917

[16] Trune DR Zheng QY. Mouse models for human otitis media. Brain Res. (2009) 1277:90–103. 10.1016/j.brainres.2009.02.047 19272362 PMC2832702

[17] Yang B Tian C Zhang Z Han F Azem R Yu H. Sh3pxd2b mice are a model for craniofacial dysmorphology and otitis media. PLoS ONE. (2011) 6:e22622. 10.1371/journal.pone.0022622 21818352 PMC3144925

[18] Tian C Yu H Yang B Han F Zheng Y Bartels CF. Otitis media in a new mouse model for CHARGE syndrome with a deletion in the Chd7 gene. PLoS ONE. (2012) 7:e34944. 10.1371/journal.pone.0034944 22539951 PMC3335168

[19] Suryani S Dharma A Nasir N. Isolation and identification of pathogenic bacteria secretion of chronic suppurative otitis media patients. Rasayan J Chem. (2018) 11:1139–43. 10.31788/RJC.2018.1131966

[20] Hayashimoto N Yasuda M Goto K Takakura A Itoh T. Study of a Bordetella hinzii isolate from a laboratory mouse. Comp Med. (2008) 58:440–6. 10.1186/1746-6148-4-39 19004369 PMC2707133

[21] Lim DJ Birck H. Ultrastructural pathology of the middle ear mucosa in serous otitis media. Ann Otol Rhinol Laryngol. (1971) 80:838–53. 5127754 10.1177/000348947108000611

[22] Fons JM Milmoe NJ Dack MRG Joshi L Thompson H Tucker AS. The interconnected relationships between middle ear bulla size, cavitation defects, and chronic otitis media revealed in a syndromic mouse model. Front Genet. (2022) 13:933416. 10.3389/fgene.2022.933416 36299576 PMC9590451

[23] Zhang Y Yu H Xu M Han F Tian C Kim S. Pathological features in the LmnaDhe/+ mutant mouse provide a novel model of human otitis media and laminopathies. Am J Pathol. (2012) 181:761–74. 10.1016/j.ajpath.2012.05.031 22819531 PMC3432432

[24] Cunsolo E Marchioni D Leo G Incorvaia C Presutti L. Functional anatomy of the Eustachian tube. Int J Immunopathol Pharmacol. (2010) 23:4–7. 20152070

[25] Netto LFS da Costa SS Sleifer P Braga MEL. The impact of chronic suppurative otitis media on children's and teenagers' hearing. Int J Pediatr Otorhinolaryngol. (2009) 73:1751–6. 10.1016/j.ijporl.2009.09.033 19853931

[26] Saunders J Murray M Alleman A. Biofilms in chronic suppurative otitis media and cholesteatoma: scanning electron microscopy findings. Am J Otolaryngol. (2011) 32:32–7. 10.1016/j.amjoto.2009.09.010 20036033

[27] Norris CR. Primary Ciliary Dyskinesia[J]. Am J Respir Crit Care Med. (2004) 169:459–67. 14656747 10.1164/rccm.200303-365OC

[28] Elsheikh MN Mahfouz ME. Up-regulation of MUC5AC and MUC5B mucin genes in nasopharyngeal respiratory mucosa and selective up-regulation of MUC5B in middle ear in pediatric otitis media with effusion. Laryngoscope. (2006) 116:365–9. 10.1097/01.MLG.0000195290.71090.A1 16540890

[29] Lei CQ Wu X Zhong X Jiang L Zhong B Shu HB. USP19 inhibits TNF-α-and IL-1β-triggered NF-κB activation by deubiquitinating TAK1. J Immunol. (2019) 203:259–68. 10.4049/jimmunol.1900083 31127032

[30] Kouskoura T Fragou N Alexiou M John N Sommer L Graf D. The genetic basis of craniofacial and dental abnormalities. Schweiz Monatsschr Zahnmed. (2011) 121:636–46. 10.5167/uzh-50358 21861247

[31] Bonser LR Erle DJ. Airway mucus and asthma: the role of MUC5AC and MUC5B. J Clin Med. (2017) 6:112. 10.3390/jcm6120112 29186064 PMC5742801

