# Corrigendum: Role of a novel mouse mutant of the *Galnt2*^*tm*1*Lat*/*tm*1*Lat*^ gene in otitis media

**DOI:** 10.3389/fneur.2023.1287032

**Published:** 2023-10-09

**Authors:** Weijun Ma, Heng Li, Juan Hu, Ying Gao, Hui Lv, Xiaotong Zhang, Qing Zhang, Min Xu, Ying Cheng

**Affiliations:** ^1^Department of Otolaryngology-Head and Neck Surgery, Second Affiliated Hospital of Xi'an Jiaotong University, Xi'an, Shaanxi, China; ^2^Department of Otorhinolaryngology, Shiquan County Hospital, Ankang, Shaanxi, China; ^3^Department of Otolaryngology-Head and Neck Surgery, Xinhua Hospital Affiliated to Shanghai Jiao Tong University School of Medicine, Shanghai, China

**Keywords:** genetic susceptibility, otitis media, mutant *Galnt2* homozygote, hearing loss, mouse model

In the published article, there was an error. In the section of “Mouse husbandry and genotyping”, some statements should be added to clarify the contributions of Dr. Tabak and his lab.

A correction has been made to the section of “Mouse husbandry and genotyping”. This paragraph previously stated:

Galnt2^tm1Lat/tm1Lat^ homozygous mice were obtained from and bred at the Wolstein Animal Research Facility of the Case Western Reserve University. A total of 64 homozygous Galnt2^tm1Lat/tm1Lat^ mutant and 60 wild-type mice were used in the present study. Mice were raised in a ventilated room with a 12-h light/dark cycle and given free access to food at 21°C. Mice of < 7 days were genotyped by the polymerase chain reaction (PCR), and the experimental protocol was approved by the Health Sciences Institutional of Animal Care Center and the Ethics Committee of Case Western Reserve University (approval numbers: 2008-0174 and 2008-0156) and the Second Affiliated Hospital of Xi'an Jiaotong University (approval number: 2019-268). The PCR primers used for tail snip genotyping are presented as follows:

P1: GGTCCTGACCTTCCTAGACAGTCACTGC

P2: GCACTCTCCAAGGGCATGACAGAGC

P3: GGGGGAGGATTGGGAAGACAATAGC

The corrected paragraph appears below:

Galnt2^tm1Lat/tm1Lat^ homozygous mice were obtained from and bred at the Wolstein Animal Research Facility of Case Western Reserve University. Sixty-four homozygous Galnt2^tm1Lat/tm1Lat^ mutant and 60 wild type mice were used in the study. Mice were raised in a ventilated room with 12-h light/dark cycle and free access to food at 21°C. Mice < 7 days were genotyped by PCR, the experimental protocol was approved by the Health Sciences Institutional of Animal Care center and Ethics Committee of Case Western Reserve University (approval numbers: 2008-0174 and 2008-0156) and Second Affiliated Hospital of Xi'an Jiaotong University (approval number: 2019-268). PCR primers used for tail snip genotyping are presented as follows:

P1: GGTCCTGACCTTCCTAGACAGTCACTGC

P2: GCACTCTCCAAGGGCATGACAGAGC

P3: GGGGGAGGATTGGGAAGACAATAGC

The mice used in this study (termed Galnt2^tm1Lat/tm1Lat^) were constructed, initially characterized, and provided by Dr. Lawrence A. Tabak, Section on Biological Chemistry, National Institute of Dental and Craniofacial Research, NIH, to Dr. Q. Zheng while he was on the faculty of CWRU. Details about the construction and characterization of these mice by Dr. Tabak and collaborators (reported as Galnt2^−/−^ mice) may be found in Verzijl et al. (11).

All information in Figure 1 was derived from data provided by Dr. Tabak and his colleagues at NIDCR, NIH. However, neither Dr. Tabak nor any member of his lab were involved in the conduct or interpretation of any remaining experiments reported in this paper. We had difficulty breeding these mice, and therefore did not use conventional back crossing prior to performing the analyses they reported.

The authors apologize for this error and state that this does not change the scientific conclusions of the article in any way. The original article has been updated.
